# Marine Genomics: A clearing-house for genomic and transcriptomic data of marine organisms

**DOI:** 10.1186/1471-2164-6-34

**Published:** 2005-03-10

**Authors:** David J McKillen, Yian A Chen, Chuming Chen, Matthew J Jenny, Harold F Trent, Javier Robalino, David C McLean, Paul S Gross, Robert W Chapman, Gregory W Warr, Jonas S Almeida

**Affiliations:** 1 Medical University of South Carolina, Charleston, South Carolina, 29425, USA; 2 Departments of Biochemistry, of Biostatistics, Bioinformatics and Epidemiology. The Marine Biomedicine & Environmental Sciences Center (MBES), 221 Ft Johnson Rd., Charleston, SC 29412, USA; 3 The Marine Genomics Consortium, Hollings Marine Laboratory, 331 Fort Johnson Road, Charleston, South Carolina 29412-9110, USA; 4 South Carolina Department of Natural Resources, 331 Ft. Johnson Road, Charleston SC 29412 (SCDNR), USA

## Abstract

**Background:**

The Marine Genomics project is a functional genomics initiative developed to provide a pipeline for the curation of Expressed Sequence Tags (ESTs) and gene expression microarray data for marine organisms. It provides a unique clearing-house for marine specific EST and microarray data and is currently available at .

**Description:**

The Marine Genomics pipeline automates the processing, maintenance, storage and analysis of EST and microarray data for an increasing number of marine species. It currently contains 19 species databases (over 46,000 EST sequences) that are maintained by registered users from local and remote locations in Europe and South America in addition to the USA. A collection of analysis tools are implemented. These include a pipeline upload tool for EST FASTA file, sequence trace file and microarray data, an annotative text search, automated sequence trimming, sequence quality control (QA/QC) editing, sequence BLAST capabilities and a tool for interactive submission to GenBank. Another feature of this resource is the integration with a scientific computing analysis environment implemented by MATLAB.

**Conclusion:**

The conglomeration of multiple marine organisms with integrated analysis tools enables users to focus on the comprehensive descriptions of transcriptomic responses to typical marine stresses. This cross species data comparison and integration enables users to contain their research within a marine-oriented data management and analysis environment.

## Background

Large collections of ESTs enable the assembly of nucleotide sequence contigs and, if the genomic sequence is available, a means of mapping the species genome and ultimately assist in gene and pathway discovery. Multivariate statistical analysis of these collections is used for microarray design. The main feature of the Marine Genomics project is that all data is accessible to the public (non-curator viewers) as a curated clearing house for genomic and transcriptomic data of marine organisms. Accordingly, Marine Genomics includes a tool for automated EST submission to NCBI's GenBank to assist in integrating data and annotation results with a wider public resource. One of the primary goals of the clearing-house is to ensure the successful submission to NCBI, of all processed and curated data contained in the Marine Genomics databases. New species databases are welcomed in order to build a comprehensive marine repository. The conglomeration of multiple marine organisms with integrated analysis tools enables focusing on the comprehensive descriptions of transcriptomic responses to typical marine stresses such as water pollution and algal blooms, effects of climate change such as altered pH and increase in carbon dioxide levels [1,2], as well as localized phenomena such as coral bleaching [3] and viral infections (crustacean and fisheries diseases [4]).

## Construction and content

### Methodology

The marine genomics pipeline is a web-based software environment. The open-source public license for the scripting language PHP 4.2.2 is used at the front end for user interface development and runs on Apache web server 1.3.33. MATLAB version 6.5 , a scientific engineering computational language, release 13 is employed for statistical data analysis and some of the more intensive computational processes. The open source database application PostgreSQL, version 7.2.3 , is used for all data storage. The entire system is developed and run on servers configured with the open source operating system Red Hat Linux version 7.3 .

### Site divisions and support functions

The site functionally divides in two main upload functions for both EST and microarray data upload. These upload tools enable users to add EST and microarray data to their species PostgreSQL database where they are accessed and manipulated by various processing tools. The microarray upload also allows users to warehouse microarray MIAME and MAGE compliant data. Other site support functions include an EST annotative text search, a stand-alone BLAST [5] function as well as user authentication and curation control.

### Species databases

Marine Genomics currently contains 19 different marine species databases. Species currently included are: *Anas platyrhynchos *(mallard), *Crassostrea gigas *(Pacific oyster), *Callinectes sapidus *(blue crab), *Crassostrea virginica *(eastern oyster), *Eubalaena glacialis *(Northern Atlantic right whale), *Fundulus *species (killifish), *Homarus americanus *(American Atlantic lobster), *Karenia brevis *(red tide algae), *Leucoraja erinacea *(little skate), *Litopenaeus setiferus *(white shrimp), *Litopenaeus stylirostris *(blue shrimp), *Litopenaeus vannamei *(white shrimp), *Montastraea annularis *(lobed star coral), *Oculina varicosa *(stony coral), *Porites porites *(clubbed finger coral), *Palaemonetes pugio *(daggerblade grass shrimp), *Squalus acanthias *(spiny dogfish) and *Tursiops truncatus *(bottlenose dolphin). Each of these species databases undergoes a cross-BLAST for sequence similarities. New databases are added regularly upon user request.

### Database Updates

In order to maintain the most current BLAST results both from GenBank and the internal Marine Genomics database, the databases will be BLAST on a quarterly basis.

## Utility

### EST pipeline implementation

#### EST Upload

Users can reference the Marine Genomics Process flow guide on the homepage of the website to get an overview of Marine Genomics EST and microarray processes. Currently the pipeline accepts both FASTA and text sequence files as well as electropherogram trace files. The user interface allows the user to upload sequences both as zipped batches and as individual uploads. The phred and the phd2fasta programs [6,7] are used for converting the trace file into a readable text format. The files are then stored for back-up. Once submitted to the pipeline each sequence undergoes a number of QA/QC procedures and subsequently becomes available for curation and user initiated submission to NCBI's GenBank.

#### EST quality control and sequence processing (QA/QC)

1. Cross-match [6] is employed to mask vector content from the uploaded sequence files. Then this masked vector is automatically removed by a Marine Genomics trimming tool.

2. The collars (user specified regions of the vector adapters) chosen by the user on file upload are used for a final vector screening in an attempt to ensure all vector is removed from the sequence before submission to the species databases. It allows the user some control in specifying the end of the vector sequence and thus adds an extra layer of vector screening and removal.

3. Poly-A tail removal.

4. Size control: Sequences shorter than 50 nucleotide bases are flagged.

5. N-content control: Flagging of sequences with an N-content of greater than 3 bases in 10.

6. Flagging of non-DNA sequences to prevent any possible file upload contamination.

#### EST curation and submission to NCBI

1. Sequence viewing: Public viewers have access to the sequence, BLAST and processing results such as when the sequence was updated and last modified as well as tissue and NCBI accession number.

2. Sequence curation: Curators can review/edit trimmed ESTs as well as delete and submit the individual sequence to GenBank.

3. Automated BLAST: Sequences automatically undergo a BLAST against NCBI's GenBank using the BLAST tool. GenBank databases currently used include the GenBank non-redundant CDS translations protein databases (nr) with BLASTx, the GenBank non-redundant nucleotide databases (nt) with BLASTn and the GenBank EST databases with BLASTn (dbEST). The sequences also undergo BLAST against all local Marine Genomics databases.

4. Interactive sequence submission to GenBank: Once each curator-uploaded sequence batch has been appropriately run through the QA/QC process, the curator receives an automated email allowing them to submit their ESTs to GenBank's dbEST database. The email also lists which sequences will not be included in the submission due to problem-flagging. The curator has the option of reviewing sequences that may have been left out of the submission process, such that they can be edited appropriately and then later submitted.

5. Contigs are assembled using CAP3 program [8] after ESTs have been curated. This enables the users to determine unique transcripts/genes. Currently curators can request the addition of this functionality for their particular species and the contig data is made web accessible then.

6. Sequences are annotated with Gene Ontology terms by searching the sequences against the Gene Ontology database [9] using BLAST [10]. Species-specific Gene Ontologies (GO) are currently reported in a piechart on each species entry page and also within each EST sequence page. Also a cross species GO data summary is reported along side the listing of all current Marine Genomics species. The addition of GO annotation is currently on going.

### Microarray pipeline implementation

The microarray pipeline is a more recent addition to Marine Genomics than the EST pipeline and is still undergoing development particularly in the buildup of analysis tools. The MATLAB statistics and bioinformatic libraries are of critical importance for the fast development and deployment of advanced analysis procedures. Accordingly, a suite of multivariate statistical analysis tools, developed in MATLAB is being employed to assist in microarray design. An optimal cDNA microarray probe selection algorithm combining different clustering methods and contig information was implemented to assist microarray design [10]. The procedure can also be used for multiple species microarray design which critically benefits from the marine-specific nature of the EST databases maintained. EST probe selection for microarray design is available upon request and the clustering and probe selection output is available on the website on each species entry page.

A MIAME compliant [11] excel template is provided for user download  to ensure the data remains compliant. This file can be filled-in and exported from Microsoft Excel as a tab-delimited MIAME data file for upload alongside the corresponding MAGE data file. Currently Marine Genomics accepts MAGE data from in-house microarray experiments run at the Hollings Marine Laboratory in Charleston, South Carolina for storage and analysis.

#### Microarray data upload and warehousing of experimental results data

1. Data upload: The microarray pipeline accepts a text file (MIAME compliant) which contains information specific to the experimental information (lab notes etc.) It also accepts a text data file, output from the microarray laser scanner, (containing specific spot locations and intensities) which is parsed into MAGE compliant data for warehousing.

2. Data warehousing: The uploaded tab-delimited files are parsed and stored in a relational PostgreSQL database. Currently warehoused microarray data is accessible to the public through specific species links.

3. Data download: Microarray data can be accessed and downloaded as Web pages or as Excel compatible files. Marine Genomics also includes a MATLAB centric microarray access feature consisting of an m-file, mgma_get.m. This function will automatically list all available microarray data and download complete microarray content directly into the user's MATLAB workspace. This makes an entire microarray dataset or selection of datasets available in a MATLAB Bioinformatics Toolbox specific format for analysis by the functions within the Bioinformatic Toolbox (e.g. maimage(mgma_get(15), Ch1 Intensity)). This m-file is made freely available at: 

## Conclusion

The Marine Genomics infrastructure was developed as a clearing house of functional genomics data for marine organisms. It currently includes tools to upload, preprocess, cross-reference, annotate, NCBI submit and store EST data. It also includes a corresponding microarray design, upload and storage tool with development of analysis tools underway.

The usage of the Marine Genomics infrastructure has been speedily increasing with more species databases being added and the numbers of sequences increasing even faster. Furthermore, Marine Genomics integrates the microarray entries with the MATLAB environment for which there are several commercial and public libraries ("toolboxes") for microarray analysis.

Current development goals include the added functionality of continued addition of ontology and contig information for all species. Another goal is to add the ability to parse and process multiple microarray platforms such that users can have the flexibility of uploading data output from their own individual microarray platforms. Finally, in particular special care will be given to speedily exporting expression data as MAGE-XML for incorporation in NCBI's GEO databases rather than having that data exclusively retained in Marine Genomics.

The ultimate purpose of Marine Genomics is indeed to assist in submitting quality data to the NCBI GenBank and GEO databases. For that purpose, the Marine Genomics pipeline and tools have been assembled to provide a medium for working with functional genomics in a marine biology environment.

### Availability and contacts

**Figure 1:**
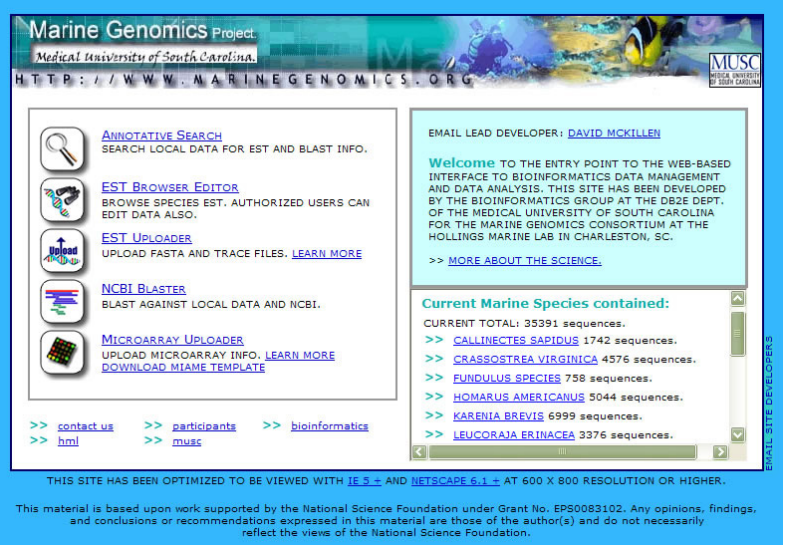
Marine Genomics homepage

The species databases are available for public viewing and new species additions can be made via the website (Fig. 1). The website URL is . Each species database is available for download in the form of a fasta file from each of the species data pages. Contact: David J. McKillen at mckilldj@musc.edu.

## Authors' contributions

DJM is the lead developer on the pipeline construction and maintenance, YAC develops the bioinformatic algorithms, CC helps with server management, MJJ and JR advise on the biological aspects of pipeline development, HFT assists in the development of the pipeline, DCM co-developed the microarray database model, PSG and RWC are responsible for project overview and management, GWW and JSA proposed the underlying conceptual model and oversee the development. All authors read and approved the final manuscript.
